# Testing the Translational Potential of Rapamycin on Healthy Aging

**DOI:** 10.1093/geroni/igaf122.406

**Published:** 2025-12-31

**Authors:** Adam Konopka

**Affiliations:** University of Wisconsin-Madison

## Abstract

Treatment with rapamycin, an inhibitor of the mechanistic Target Of Rapamycin Complex One (mTORC1) protein kinase, has been repeatedly demonstrated to extend lifespan and prevent or delay several age-related diseases in diverse model systems. Concerns over the risk of adverse side effects, including immunosuppression and metabolic disruptions, have cautiously limited the clinical translation of rapamycin and its analogs as a treatment for aging associated conditions. Despite potential risks, an increasing number of physically active adults are taking the mTOR inhibitor rapamycin off label with the goal of extending healthspan. A working model suggests that while inhibition of mTORC1 promotes healthy aging, many of the negative side effects of rapamycin are associated with inhibition of a second mTOR complex, mTORC2. Differences in the kinetics and molecular mechanisms by which rapamycin inhibits mTORC1 and mTORC2 suggest that a therapeutic window for rapamycin could be exploited using intermittent dosing schedules or alternative rapalogs that may enable more selective inhibition of mTORC1. However, the optimal dosing schedules and the long-term efficacy of such interventions in humans is unknown. Further, it remains unknown how different rapamycin dosing schedules impact the health benefits of exercise. Here, I will provide the rationale, experimental design, and updates for two ongoing human clinical trials aimed to determine the safety, pharmacokinetics, pharmacodynamics, and efficacy of rapamycin and rapalogs on several clinically oriented outcomes. I will also share new insights from integrated multi-omics studies that reveal the impact of different rapamycin dosing schedules on the molecular and metabolic adaptations to exercise in older mice. Results from these pre-clinical and early phase studies will help guide the design of future clinical trials to determine whether rapamycin can be used safely to inhibit mTORC1 for the treatment and prevention of age-related diseases in humans.

